# Inheritance of Calyx Abscission in Apple: A Trait with Potential Impact on Fruit Rot Susceptibility

**DOI:** 10.3390/plants14233674

**Published:** 2025-12-02

**Authors:** Matthias Pfeifer, Andreas Peil, Henryk Flachowsky, Thomas Wöhner

**Affiliations:** 1 Julius Kühn-Institut (JKI)—Federal Research Centre for Cultivated Plants, Institute for Breeding Research on Fruit Crops, 01326 Dresden-Pillnitz, Germany; matthias.pfeifer@julius-kuehn.de (M.P.); andreas.peil@julius-kuehn.de (A.P.); henryk.flachowsky@julius-kuehn.de (H.F.); 2 Institute of Plant Genetics, Department of Molecular Plant Breeding, Leibniz University Hannover, 30419 Hannover, Germany

**Keywords:** apple, calyx abscission, inheritance, fruit rot susceptibility, wild *Malus* species, disease resistance, QTL analysis

## Abstract

Fruit rots, both pre- and postharvest, represent a major problem in apple production, leading to significant yield losses each year. In this study, the inheritance of calyx abscission, a trait that could potentially reduce susceptibility to various fruit rots, was investigated in an F_1_ population. Calyx persistence rates were phenotyped in the field in 2023 and 2025 on 122 offspring derived from a cross between ‘Idared’ and *Malus baccata* ‘Jackii’, the latter exhibiting complete calyx abscission. QTL analyses were conducted using genotypic data and a genetic linkage map generated in a previous study. Results show, for the first time in apple, that calyx abscission is a heritable trait influenced by multiple loci, with the strongest effects detected on linkage groups 5 and 13. Whether calyx abscission is linked to reduced susceptibility to fruit rots, and for which pathogens this applies, remains to be investigated in future studies.

## 1. Introduction

Cultivated apples (*Malus domestica* Borkh.) are among the most economically important fruit crops, and their ability to be stored for several months in storage rooms allows them to be marketed at the desired time and in desired quantities, thereby securing a year-round supply [1]. During fruit development and postharvest storage, a wide range of pathogens can cause fruit rots, potentially leading to significant losses [1,2,3,4,5,6]. The occurrence of these pathogens exhibits variation both from year to year and between different regions [2,4,5]. For instance, in Northern Germany, a five-year survey revealed that *Botrytis cinerea* and *Neonectria ditissima* were most frequently detected on blossom-end rot fruits, with 41.6% and 33.9% prevalence, respectively, though the dominant pathogen varied between years [5]. In some cases in Washington State, *B. cinerea*, *Sphaeropsis pyriputrescens*, and *Phacidiopycnis washingtonensis* were responsible for losses exceeding 20% [1]. In Chile, up to 46% of fruits were found to be infected with mouldy core, with *Alternaria* species accounting for 67.7% of the isolated fungi [6]. Meanwhile, in China, up to 70% of fruit harvested from certain orchards exhibited signs of mouldy core and core rot, with the latter being most frequently caused by *Trichothecium roseum* [4]. As diverse as the pathogens are, so too are the possible modes of fruit infection. Latent infections in the field, as well as infections during and after harvest, such as during handling and packing, can occur through blossoms, natural openings like stomata, lenticels, open sinuses (connection between core and calyx tube), or the stem, as well as through wounds or cracks [2,4]. The presence of a wide variety of pathogens and infection mechanisms has led to a situation in which cultivars exhibit resistance to some pathogens but susceptibility to others [7]. This makes breeding for universal fruit rot resistance highly challenging. Despite the fact that the exact timing and mode of infection are still unclear for many pathogens, the calyx region often appears to play a crucial role. Rots frequently originate there, and sporulation on sepals, for example by *B. cinerea*, has been observed [5]. In specific cultivars, such as ‘Red Delicious’ and ‘Fuji’, sepals appear to be the primary infection sites for *S. pyriputrescens* [8]. Consequently, the breeding of apples with a detached calyx has the potential to reduce susceptibility to various fruit rots and may also hinder sporulation if sepals are no longer present. Despite the fact that apples displaying calyx abscission will not be resistant to all fruit rot pathogens, we hypothesise that this trait has the potential to generally reduce the risk of multiple fruit rots simultaneously. This outcome may be attributed to a number of factors, including the reduced probability of infection via sepals (which are shed after flowering), the limited establishment and spread of pathogens due to the absence of flower debris, and the faster drying process and a cleaner surface at the site of calyx detachment. Furthermore, it should be mentioned that the persistence or detachment of the calyx has also taxonomical relevance, as it represents an important diagnostic trait used in identification keys within the complex genus *Malus* [9,10]. The objective of this study was therefore to investigate the inheritance of calyx abscission in apple, as a preliminary step toward understanding its potential role in fruit rot resistance. For this purpose, genotypic and phenotypic data from 122 F_1_ individuals of the cross population ‘Idared’ × *Malus baccata* ‘Jackii’ (*Mb*j) were utilised. The latter produces only fruits with a deciduous calyx, a trait that is uncommon in cultivated apples and sometimes observed in accessions of wild *Malus* species such as *M. baccata*, *M. halliana*, *M. hupehensis*, *M. rockii*, *M. sikkimensis*, *M. spontanea* and others [9], though, to the knowledge of the authors, not at all in cultivars grown for commercial fruit production.

## 2. Results

### 2.1. Phenotypic Variation in Calyx Abscission Among F_1_ Individuals

**Figure 1:**
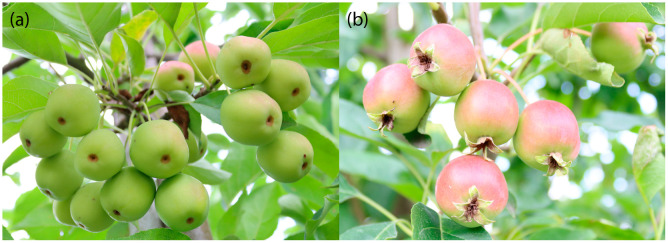
F_1_ individuals producing fruits with (**a**) detached calyces and (**b**) persistent calyces only.

In 2023, among the 122 F_1_ individuals, 28 exhibited complete calyx abscission, 61 displayed a fully persistent calyx, and 31 produced fruits both with and without a detached calyx (12 mostly with detached calyces, 19 mostly with persistent calyces). For two genotypes, no fruits were available for phenotyping. In 2025, the results were slightly different: 23 individuals showed complete calyx abscission, 42 displayed complete persistence, and 54 produced both fruit types, with an average of 4.83 persistent calyces out of ten fruits assessed. For three genotypes, no fruits could be phenotyped. The detailed results for both years are presented in Table S1. During phenotyping, it was further observed that in genotypes with both fruit types, fruits developing in shaded areas of the tree tended to exhibit calyx abscission more frequently than those exposed to direct sunlight. The Spearman correlation coefficient between the two years was 0.85, indicating a high degree of consistency. Figure 1 shows representative fruits from two F_1_ individuals, one with complete calyx abscission and one with a persistent calyx.

### 2.2. Linkage Mapping of Calyx Abscission in Mbj

**Figure 2:**
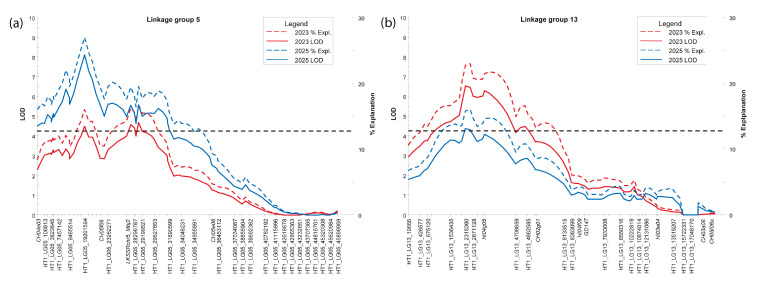
Logarithm of odds (LOD) score profiles and corresponding percentages of explained variance along linkage groups 5 (**a**) and 13 (**b**) of *Malus baccata* ‘Jackii’ haplotype 1. The genome-wide significance threshold of 4.3, shown as a dashed line, was calculated using permutation testing with 1000 iterations at a 95% confidence level.

Quantitative trait loci (QTLs) associated with calyx abscission in *Mb*j were identified on linkage group (LG) 5 and LG 13, exceeding the genome-wide significance threshold of 4.3 in both 2023 and 2025. On LG 5 (Figure 2a), the highest logarithm of odds (LOD) scores were 4.69 in 2023, explaining 16.7% of the variance, and 8.10 in 2025, explaining 27.0%. On LG 13 (Figure 2b), the maximum LOD score was 6.53 in 2023 (22.7% variance explained) and 4.38 in 2025 (15.8% variance explained). Markers with the highest LOD scores on linkage groups 5 and 13 in both years showed Kruskal–Wallis (KW) test values ranging from 15.37 to 30.58 (df = 1), with *p*-values consistently below 0.0001.

In addition to the QTLs described above, further QTLs associated with calyx abscission were identified on other linkage groups. However, these were only significant at the chromosome-wide threshold and, with the exception of the one on LG 11, were significant in only one of the two assessed years. Table 1 provides an overview of the markers with the highest LOD scores for these QTLs, along with their corresponding percentages of explained variance. KW analysis of the markers with the highest LOD scores on linkage groups 3, 14 and 15 yielded significant results, with *p*-values below 0.001. In contrast, the markers HT1_LG11_2883941 and HT1_LG11_4228999 on LG 11 showed no significance in KW analysis in either 2023 or 2025, although other markers on LG 11 did show significance, with *p*-values below 0.001 in both years.

### 2.3. Calyx Abscission-Associated SNP Marker Alleles Are Located on Both Haplotypes of Mbj

Comparison of the mean calyx persistence rates for 2023 and 2025 for F_1_ individuals carrying different alleles of single-nucleotide polymorphism (SNP) markers HT1_LG05_19201594 and HT1_LG13_2315360 indicated that the calyx abscission-associated alleles are located on haplotype (HT) 2 and HT1 of *Mb*j, respectively (see Table 2). SNP marker HT1_LG05_19201594 is located at position 18,974,705 on LG 5 of HT2 in *Mb*j.

### 2.4. Association Between SNP Marker Allele Combinations and Calyx Abscission

To evaluate the effect of SNP markers HT1_LG05_19201594 and HT1_LG13_2315360 on calyx abscission, the mean calyx persistence rates of F_1_ individuals carrying different allele combinations were compared. Of the 122 F_1_ individuals, 34 carried both SNP alleles associated with calyx abscission at these markers, whereas 33 individuals carried both alleles associated with calyx persistence. The remaining 55 individuals carried only one of the two calyx abscission-associated alleles. Table 3 summarises the mean calyx persistence rates for these three groups. In 2025, individuals carrying both calyx abscission-associated SNP alleles had on average only 2.03 fruits with persistent calyx out of 10, compared to 8.71 fruits among those with both calyx persistence-associated alleles. However, among the 34 individuals carrying both calyx abscission-associated SNP marker alleles, 10 were still classified with a rating score of 2 or 3 in 2023, corresponding to a majority or all fruits with persistent calyx. In 2025, 7 of these 34 individuals produced 6 to 10 fruits with persistent calyx out of the 10 assessed.

## 3. Discussion

To our knowledge, this is the first study to investigate the inheritance of calyx abscission in apple that combines phenotypic and genotypic data. However, the inheritance of calyx abscission was already examined in the 20th century, primarily to improve the taxonomic understanding of *Malus* [11,12]. In crosses between *M. domestica* and wild *Malus* species with deciduous calyces, offspring typically exhibited three phenotypes: complete calyx persistence, complete calyx abscission, or a mixture of fruits with and without a detached calyx [11,12]. In these studies, individuals with calyx persistence or mixed fruit types were generally more frequent than those with complete calyx abscission, consistent with our observations [11,12]. Notably, Henning [11] also reported a cross between *M. zumi* and ‘Transparente de Croncels’, in which all 15 offspring exhibited only detached calyces. Based on near 3:1 segregations of calyx persistence to calyx abscission observed in other cross combinations, Henning [11] hypothesised that the inheritance of calyx abscission might be controlled by two dominant complementary genes, with wild *Malus* species, which possess a deciduous calyx, being heterozygous for both genes, and *M. domestica* being homozygous recessive. Mildenberger [12] further observed that some genotypes inherited calyx abscission more consistently than others, suggesting the existence of at least two different underlying mechanisms. He also reported that *M. baccata* seedlings displayed higher rates of calyx abscission compared to seedlings of *M. toringo* or *M. floribunda* when crossed with *M. domestica* [12].

The results of our QTL analysis support a polygenic mode of inheritance, with several loci on different linkage groups influencing calyx abscission. This is further supported by the absence of a classical 1:1 segregation pattern and the presence of genotypes in which some fruits retained the calyx while others did not. We also observed a near 3:1 segregation of full or partial calyx persistence to complete calyx abscission in our population, and QTL mapping identified two major loci on LG 5 and 13, supporting Henning’s [11] hypothesis that two genes may be involved in controlling this trait. However, calyx abscission could not be fully explained by these two loci alone, and additional loci on other linkage groups also appear to influence this trait significantly. Environmental factors, and potentially genotype-by-environment interactions, further affected calyx abscission, as phenotypic data and QTL effects varied between years. This variation may, however, be partly attributable to the use of different assessment scales. Our findings are in accordance with previous studies conducted on pears, which demonstrated that calyx abscission is influenced by a combination of genetic and environmental factors [13,14,15]. The influence of environmental factors is also supported by our observation that calyx abscission was more common in fruits growing in shaded areas than in those exposed to full sunlight. This observed effect in apple may be mediated by phytohormones. It has been described in pear that phytohormones play an important role in calyx abscission, with auxin inhibiting this process and ethylene promoting it [15,16,17,18]. However, it should be noted that species-specific differences may exist between *Malus* and *Pyrus*. In general, calyx abscission is nowadays much better studied in pear than in apple, and pears without a calyx are associated with improved fruit quality and higher market value [15,16,17,18,19,20,21,22]. In pear, a sport exhibiting higher rates of calyx abscission has even been selected, underlining the importance of this trait, though primarily due to its impact on fruit quality rather than disease resistance [18].

The inheritance of calyx abscission has also been explored in pear [21,23]. Westwood and Bjornstad [23] reported that calyx persistence is a dominant trait. In contrast, Kang et al. [21] observed that the paternal parent had a stronger influence on the trait than the maternal parent. Crosses involving a paternal parent with <10% calyx-persistent fruits and a maternal parent with >90% calyx-persistent fruits resulted in F_1_ populations in which the <10% class was the most frequent [21]. In our cross-population, the genotype *Mb*j served as the pollen donor. However, the strong paternal effect described by Kang et al. [21] was not evident in our progeny, as genotypes with persistent calyx or predominantly persistent calyx were in the majority. Future investigations are needed to clarify whether a similar paternal effect exists in *Malus*. Furthermore, analysing larger segregating populations across different environments could help refine the QTL regions reported here and enable the discovery of candidate genes responsible for calyx abscission.

We hypothesised that calyx abscission in apples might reduce the susceptibility to fruit rots, for example in ‘Red Delicious’ and ‘Fuji’ regarding *S. pyriputrescens*, as the sepals appear to be the main infection site [8]. However, for other pathogens such as *A. alternata*, where infection can reach the ovary seven days after full bloom, calyx abscission might have no effect, with other calyx-independent resistance factors potentially playing a more prominent role [3]. That said, the influence of calyx abscission on fruit rot susceptibility needs to be verified individually for each pathogen in future studies. Genotypes producing fruits with both detached and persistent calyces are particularly suitable for these studies, as they allow the effect of calyx abscission to be assessed while excluding genetic factors. It should also be considered that apple rots are often caused by a complex of different pathogens [4,6]. It has been shown that during fruit development, when the sepals close and flower debris becomes trapped within the calyx tube, pathogens such as *T. roseum* can persist there, potentially serving as inoculum and enabling later core infections when an open sinus forms [4]. Consequently, abscission of the calyx in combination with the removal of flower debris has the potential to reduce the presence of such pathogens and ultimately result in a cleaner fruit surface and reduced fruit rots. Furthermore, bacterial pathogens such as *Erwinia amylovora*, which have previously led to import bans on apples, can also persist in the calyx, and calyx abscission is therefore likely to reduce the presence of *E. amylovora* as well [24,25].

In the present F_1_ population, calyx abscission was observed shortly after or simultaneously with blossom drop. Nevertheless, differences were observed, as in some genotypes the dead calyx remained attached to the fruit, whereas in others it did not. This observed difference could represent an important distinction with regard to susceptibility to fruit rots. Given that calyx abscission is heritable from *Mb*j, a wild apple genotype with poor fruit quality, substantial breeding effort would be required to introgress this trait into modern cultivars [26]. The fact that it is controlled by more than one gene and influenced by environmental factors makes the breeding process even more challenging. However, when *Mb*j is used in resistance breeding, for example against apple scab [27], powdery mildew [28,29], fire blight [30,31] or apple blotch [32], calyx abscission could be considered as an additional trait for selection. Notably, the use of SNPs such as HT1_LG05_19201594 and HT1_LG13_2315360 enables the selection of genotypes with higher calyx abscission without phenotypic data. However, a considerable proportion of individuals (approximately 20–30%) will still exhibit little or no calyx abscission, even when carrying the calyx abscission-associated alleles. Therefore, it should first be clarified whether fruits with detached calyces indeed show reduced susceptibility to fruit rots, in order to determine whether the development of molecular markers and the introgression of this trait are worthwhile in apple breeding. Potential effects on consumer preferences should also be considered, as alterations in fruit morphology may influence market acceptance.

## 4. Materials and Methods

### 4.1. Plant Material and Calyx Persistence Assessment

In total, 122 F_1_ individuals derived from a cross between ‘Idared’ and *Mb*j were cultivated in the experimental field at the Julius Kühn-Institut (JKI) in Dresden-Pillnitz, Germany, along with both parents. In late September 2023, the degree of calyx persistence was assessed using the ordinal scale shown in Table 4, which provided a categorical evaluation of the trait. In 2024, no assessment could be conducted due to a severe spring frost event that resulted in a complete lack of fruit development. In early July 2025, the F_1_ population was phenotyped a second time. On this occasion, a more detailed phenotyping approach was applied, in which ten fruits per genotype were individually examined, and the number of fruits with persistent calyx was counted, allowing calyx persistence to be quantified on a metric scale from 0 to 10. The phenotyping in 2023 and 2025 was performed directly on fruits on the tree by a single evaluator, and both assessments reflect the same underlying trait, namely the proportion of fruits retaining the calyx. This makes the two scoring scales directly comparable. Notably, in both the 2023 ordinal and 2025 metric scales, higher scores correspond to a higher degree of calyx persistence, i.e., lower calyx abscission. The Spearman correlation coefficient was subsequently calculated to evaluate the consistency of calyx persistence rates between 2023 and 2025.

### 4.2. Genotyping, Genetic Linkage Map Construction, and Trait-Marker Association Analyses

The genotypic data and genetic linkage map used in this study were produced in prior studies [29,33]. Briefly, the F_1_ population and both parents were genotyped using tunable genotyping-by-sequencing [34] in combination with simple sequence repeat (SSR) markers. SNPs were identified by aligning the sequences to HT1 of the *Mb*j reference genome [33], followed by quality filtering and imputation of missing values. Subsequently, SNP and SSR markers were used to construct a genetic linkage map with JoinMap 5 [35], applying the regression mapping algorithm and the Kosambi mapping function. This genetic linkage map, together with the genotypic and phenotypic data, served as the basis for the trait-marker association analyses conducted in this study using MapQTL 5 [36]. Markers significantly linked to calyx abscission were identified using KW testing, and QTLs were detected by simple interval mapping without cofactors. Genome-wide and chromosome-wide significance thresholds were determined via permutation testing with 1000 iterations at a 95% confidence level.

### 4.3. Assignment of Calyx Abscission-Associated SNP Marker Alleles to Haplotypes of Mbj

SNP marker sequences with the highest LOD scores on LG 5 and LG 13, namely HT1_LG05_19201594 and HT1_LG13_2315360, were aligned to HT1 and HT2 of the *Mb*j genome [33] using the Basic Local Alignment Search Tool [37] in CLC Main Workbench 25.0 (Qiagen, Venlo, The Netherlands). This allowed the identification of the corresponding SNP alleles on each haplotype. Subsequently, the 122 F_1_ individuals were grouped according to their alleles at each SNP marker, and the allele associated with increased calyx abscission was defined as the one present in the group with the higher mean number of detached calyces. SNP alleles of ‘Idared’ for HT1_LG05_19201594 and HT1_LG13_2315360 were previously determined by tunable genotyping-by-sequencing.

## Figures and Tables

**Table 1:** Overview of QTLs significant only at the chromosome-wide level.

Assessment Year	LG	Marker with Highest LOD Score	LOD Score	Chromosome-Wide Significance Threshold	Explained Phenotypic Variance (%)
2023	3	HT1_LG03_34571701	4.20	3.3	14.9
2023	11	HT1_LG11_2883941	3.31	2.8	14.7
2025	11	HT1_LG11_4228999	2.86	2.7	10.6
2025	14	HT1_LG14_24253769	2.21	2.2	8.2
2025	15	HT1_LG15_3889288	3.44	2.8	12.5

LG: linkage group; LOD: logarithm of odds; QTL: quantitative trait locus.

**Table 2:** Mean calyx persistence rates of F_1_ individuals for SNP markers HT1_LG05_19201594 and HT1_LG13_2315360, showing the calyx abscission-associated haplotypes of *Mb*j.

SNP Marker	SNP-Alleles in ‘Idared’	SNP-Allele in *Mb*j HT1	SNP-Allele in *Mb*j HT2	Assessment Year (Scale Type)	Mean Calyx Persistence Rate of Heterozygous F_1_	Mean Calyx Persistence Rate of Homozygous F_1_
HT1_LG05_19201594	TT	T	G	2023 (ordinal)	1.40	2.37
HT1_LG05_19201594	TT	T	G	2025 (metric)	3.35	7.62
HT1_LG13_2315360	GG	A	G	2023 (ordinal)	1.45	2.57
HT1_LG13_2315360	GG	A	G	2025 (metric)	4.34	7.50

HT: haplotype; *Mb*j: *Malus baccata* ‘Jackii’; SNP: single-nucleotide polymorphism.

**Table 3:** Mean calyx persistence rates of F_1_ individuals grouped by SNP marker allele combinations of HT1_LG05_19201594 and HT1_LG13_2315360.

Assessment Year (Scale Type)	Mean Calyx Persistence Rate of F_1_ Individuals with Both Calyx Abscission-Associated SNP Alleles	Mean Calyx Persistence Rate of F_1_ Individuals with One Calyx Abscission-Associated SNP Allele	Mean Calyx Persistence Rate of F_1_ Individuals with no Calyx Abscission-Associated SNP Alleles
2023 (ordinal)	0.88	2.12	2.75
2025 (metric)	2.03	6.20	8.71

**Table 4:** Ordinal scale for evaluating calyx persistence rate in 2023.

Rating Score	Description
0	100% of fruits with detached calyx
1	Majority of fruits with detached calyx
2	Majority of fruits with persistent calyx
3	100% of fruits with persistent calyx

## Data Availability

Data is contained within the article or Supplementary Material.

## Supplementary Materials

The following supporting information can be downloaded at: https://www.mdpi.com/article/10.3390/plants14233674/s1, Table S1: Overview of calyx persistence scores for the 122 F_1_ individuals.

## Abbreviations

The following abbreviations are used in this manuscript:

LG	linkage group
LOD	logarithm of odds
*Mb*j	*Malus baccata* ‘Jackii’
KW	Kruskal–Wallis
HT	haplotype
SNP	single-nucleotide polymorphism
SSR	simple sequence repeat
QTL	quantitative trait locus

## References

[1] Xiao C.L., Kim Y.K. (2008). Postharvest Fruit Rots in Apples Caused by *Botrytis cinerea*, *Phacidiopycnis washingtonensis*, and *Sphaeropsis pyriputrescens*. Plant Health Prog..

[2] Nybom H., Ahmadi-Afzadi M., Rumpunen K., Tahir I. (2020). Review of the Impact of Apple Fruit Ripening, Texture and Chemical Contents on Genetically Determined Susceptibility to Storage Rots. Plants.

[3] Niem J., Miyara I., Ettedgui Y., Reuveni M., Flaishman M., Prusky D. (2007). Core Rot Development in Red Delicious Apples Is Affected by Susceptibility of the Seed Locule to *Alternaria alternata* Colonization. Phytopathology.

[4] Dai P., Jiang Y., Liang X., Gleason M.L., Zhang R., Sun G. (2020). *Trichothecium roseum* Enters ‘Fuji’ Apple Cores Through Stylar Fissures. Plant Dis..

[5] Weber W.S., Dralle N. (2013). Fungi associated with blossom-end rot of apples in Germany. Eur. J. Hortic. Sci..

[6] Elfar K., Zoffoli J.P., Latorre B.A. (2018). Identification and Characterization of *Alternaria* Species Associated with Moldy Core of Apple in Chile. Plant Dis..

[7] Spotts R.A., Cervantes L.A., Mielke E.A. (1999). Variability in Postharvest Decay Among Apple Cultivars. Plant Dis..

[8] Kim Y.K., Curry E.A., Xiao C.L. (2014). Infection of apple fruit by *Sphaeropsis pyriputrescens* in the orchard in relation to Sphaeropsis rot in storage. Eur. J. Plant Pathol..

[9] Sutton J., Dunn N. (2021). ‘*Malus*’ from Trees and Shrubs Online. https://www.treesandshrubsonline.org/articles/malus/.

[10] (2025). ‘*Malus*’ from Native Plant Trust. https://gobotany.nativeplanttrust.org/dkey/malus/#c2.

[11] Henning W. (1947). Morphologisch-systematische und genetische Untersuchungen an Arten und Artbastarden der Gattung *Malus*. Der Züchter.

[12] Mildenberger G. (1963). Studien zur Taxonomie der Gattung *Malus*: I. Morphologisch-genetische Untersuchungen. Arch. Für Gartenbau.

[13] Guo G., Wei P., Yu T., Zhang H., Heng W., Liu L., Zhu L., Jia B. (2024). *PbrARF4* contributes to calyx shedding of fruitlets in ‘Dangshan Suli’ pear by partly regulating the expression of abscission genes. Hortic. Plant J..

[14] Pei M., Niu J., Li C., Cao F., Quan S. (2016). Identification and expression analysis of genes related to calyx persistence in Korla fragrant pear. BMC Genom..

[15] Yu M., Han F., Zhou N., Wang L., Li Y., Fan W., Zhang T., Bao J. (2025). Dynamics of Phytohormones in Persistent Versus Deciduous Calyx Development in Pear Revealed by Targeted Metabolomics. Horticulturae.

[16] Zheng L., Wen Y., Lin Y., Tian J., Shaobai J., Hao Z., Wang C., Sun T., Wang L., Chen C. (2024). Phytohormonal dynamics in the abscission zone of Korla fragrant pear during calyx abscission: A visual study. Front. Plant Sci..

[17] Wen Y., Shao B., Hao Z., Wang C., Sun T., Han Y., Tian J., Zhang F. (2024). Preliminary Study on Programmed Cell Death during Calyx Abscission of Korla Fragrant Pear. Horticulturae.

[18] Yang X., Wang S., Jiang Z., Zhang C., Zhao L., Cui Y. (2024). Comprehensive Physiology, Cytology, and Transcriptomics Studies Reveal the Regulatory Mechanisms Behind the High Calyx Abscission Rate in the Bud Variety of Korla Pear (*Pyrus sinkiangensis* ‘Xinnonglinxiang’). Plants.

[19] Hu H., Pan L., Sun K., Tu S., Sun Y., Wei Y., Tu K. (2017). Differentiation of deciduous-calyx and persistent-calyx pears using hyperspectral reflectance imaging and multivariate analysis. Comput. Electron. Agric..

[20] Su J., Jia B., Jia S., Ye Z.-F., Heng W., Zhu L.-W. (2015). Effect of plant growth regulators on calyx abscission, fruit quality, and auxin-repressed protein (*ARP*) gene expression in fruitlets of ‘Dangshansuli’ pear (*Pyrus bretschneideri* Rehd.). J. Hortic. Sci. Biotechnol..

[21] Kang S.-S., Kim Y.-K., Choi J.-J., Cho K.-S., Won K.-H., Lee H.C., Yu D.J., Lee H.J. (2013). Calyx Abscission in Pear (*Pyrus* spp.) Cultivars and Its Inheritance. Korean J. Hortic. Sci. Technol..

[22] Ma L., Zhou L., Quan S., Xu H., Yang J., Niu J. (2019). Integrated analysis of mRNA-seq and miRNA-seq in calyx abscission zone of Korla fragrant pear involved in calyx persistence. BMC Plant Biol..

[23] Westwood M.N., Bjornstad H.O. (1971). Some Fruit Characteristics of Interspecific Hybrids and Extent of Self- Sterility in *Pyrus*. Bull. Torrey Bot. Club.

[24] Taylor R.K., Hale C.N., Gunson F.A., Marshall J.W. (2003). Survival of the fire blight pathogen, *Erwinia amylovora*, in calyxes of apple fruit discarded in an orchard. Crop Prot..

[25] Ordax M., Biosca E.G., Wimalajeewa S.C., López M.M., Marco-Noales E. (2009). Survival of *Erwinia amylovora* in mature apple fruit calyces through the viable but nonculturable (VBNC) state. J. Appl. Microbiol..

[26] Flachowsky H., Le Roux P.-M., Peil A., Patocchi A., Richter K., Hanke M.-V. (2011). Application of a high-speed breeding technology to apple (*Malus* × *domestica*) based on transgenic early flowering plants and marker-assisted selection. New Phytol..

[27] Gygax M., Gianfranceschi L., Liebhard R., Kellerhals M., Gessler C., Patocchi A. (2004). Molecular markers linked to the apple scab resistance gene *Vbj* derived from *Malus baccata jackii*. Theor. Appl. Genet..

[28] Dunemann F., Schuster M. (2009). Genetic characterization and mapping of the major powdery mildew resistance gene *Plbj* from *Malus baccata jackii*. Acta Hortic..

[29] Pfeifer M., Kurzweg L., Muçaj B., Burkhardt T., Peil A., Flachowsky H., Emeriewen O.F., Wöhner T. (2025). Genetic mapping, marker development, and identification of candidate genes for powdery mildew resistance in *Malus baccata* ‘Jackii’. bioRxiv.

[30] Vogt I., Wöhner T., Richter K., Flachowsky H., Sundin G.W., Wensing A., Savory E.A., Geider K., Day B., Hanke M.-V. (2013). Gene-for-gene relationship in the host-pathogen system *Malus* × *robusta* 5-*Erwinia amylovora*. New Phytol..

[31] Wöhner T.W., Richter K., Sundin G.W., Zhao Y., Stockwell V.O., Sellmann J., Flachowsky H., Hanke M.-V., Peil A. (2018). Inoculation of *Malus* genotypes with a set of *Erwinia amylovora* strains indicates a gene-for-gene relationship between the effector gene *eop1* and both *Malus floribunda* 821 and *Malus* ‘Evereste’. Plant Pathol..

[32] Wöhner T., Emeriewen O.F., Höfer M. (2021). Evidence of apple blotch resistance in wild apple germplasm (*Malus* spp.) accessions. Eur. J. Plant. Pathol..

[33] Pfeifer M., Emeriewen O.F., Flachowsky H., Höfer M., Keilwagen J., Lim F.-S., Peil A., Zetzsche H., Wöhner T. (2025). High-quality haplotype-resolved genome assembly and annotation of *Malus baccata* ‘Jackii’. bioRxiv.

[34] Ott A., Liu S., Schnable J.C., Yeh C.-T.E., Wang K.-S., Schnable P.S. (2017). tGBS^®^ genotyping-by-sequencing enables reliable genotyping of heterozygous loci. Nucleic Acids Res..

[35] Van Ooijen J.W. (2018). JoinMap 5, Software for the Calculation of Genetic Linkage Maps in Experimental Populations of Diploid Species.

[36] Van Ooijen J.W. (2004). MapQTL 5, Software for the Mapping of Quantitative Trait Loci in Experimental Populations.

[37] Altschul S.F., Gish W., Miller W., Myers E.W., Lipman D.J. (1990). Basic local alignment search tool. J. Mol. Biol..

