# Supplementary material for: Inheritance of Calyx Abscission in Apple: A Trait with Potential Impact on Fruit Rot Susceptibility
Source: Plants (Basel). 2025 Dec 2;14(23):3674. doi: 10.3390/plants14233674 (PMC12694397; doi:10.3390/plants14233674)
Supplement: Supplementary file 1 [file plants-14-03674-s001.zip › Table S1.pdf]

Table S1. Overview of calyx persistence scores for the 122 F_1 individuals

Genotype	Assessment score 2023 (ordinal scale)	Assessment score 2025 (metric scale)
05225_002	3	9
05225_003	3	10
05225_005	1	2
05225_006	3	7
05225_007	NA	NA
05225_008	3	3
05225_009	0	0
05225_010	1	4
05225_011	3	10
05225_012	3	10
05225_013	1	3
05225_014	3	10
05225_017	0	1
05225_019	0	0
05225_021	1	1
05225_023	2	9
05225_024	0	0
05225_025	3	10
05225_026	3	10
05225_031	3	6
05225_032	3	10
05225_034	0	0
05225_036	2	2
05225_037	3	10
06228_002	3	10
06228_003	3	7
06228_004	0	0
06228_007	3	9
06228_008	3	10
06228_009	3	10
06228_010	3	10
06228_011	3	9
06228_012	2	9
06228_015	3	10
06228_016	0	0
06228_017	0	0
06228_018	3	8
06228_019	2	7
06228_020	3	3
06228_021	3	10
06228_022	1	3
06228_023	0	0
06228_024	2	3
06228_025	2	2
06228_026	0	0
06228_028	0	1
06228_029	0	NA
06228_030	3	1
06228_031	3	9
06228_032	3	3
06228_033	1	7
06228_034	2	9

06228_035	3	10
06228_036	0	0
06228_037	3	10
06228_039	0	0
06228_040	0	0
06228_041	3	4
06228_043	3	8
06228_044	3	6
06228_046	0	0
06228_048	2	4
06228_049	1	7
06228_050	3	10
06228_051	0	0
06228_053	2	10
06228_056	3	10
06228_058	2	6
06228_059	3	10
06228_060	2	7
06228_061	1	6
06228_062	0	1
06228_063	2	1
06228_066	2	2
06228_067	1	3
06228_068	0	0
06228_069	3	10
06228_070	3	10
06228_072	3	10
06228_075	2	4
06228_076	3	10
06228_077	0	2
06228_078	3	10
06228_079	1	0
06228_080	0	0
06228_081	3	10
06228_082	0	0
06228_084	3	10
06228_085	1	2
06228_086	3	10
06228_087	3	10
06228_088	3	10
06228_090	2	4
06228_092	2	6
06228_093	0	0
06228_095	3	10
06228_096	3	10
06228_097	0	0
06228_098	3	10
06228_099	2	3
06228_100	2	2
06228_101	3	10
06228_102	0	0
06228_103	1	0
06228_104	3	10
06228_105	0	0
06228_107	3	10
06228_110	3	10

06228_112	3	7
06228_113	3	9
06228_114	3	8
06228_115	3	10
06228_116	3	9
06228_118	3	10
06228_119	3	10
06228_120	0	1
06228_121	3	2
06228_122	3	10
06228_123	NA	NA
06228_125	2	8
06228_127	3	10
06228_128	0	2

NA: not available.
